# A network pharmacology approach to uncover the key ingredients in *Ginkgo Folium* and their anti-Alzheimer’s disease mechanisms

**DOI:** 10.18632/aging.203348

**Published:** 2021-07-27

**Authors:** Peng Zeng, Meng Fang, Han Zhao, Jing Guo

**Affiliations:** 1 Department of Pathology and Pathophysiology, School of Basic Medicine, Tongji Medical College, Key Laboratory of Neurological Disease of National Education Ministry and Hubei Province, Huazhong University of Science and Technology, Wuhan 430030, China; 2 School of Medicine, Jianghan University, Wuhan 430056, China

**Keywords:** Alzheimer’s disease, *Ginkgo Folium*, network pharmacology, genkwanin, AD pathology

## Abstract

This study aimed to identify potential anti-Alzheimer’s disease (AD) targets and action mechanisms of *Ginkgo Folium* (GF) through a network pharmacology approach. Eighty-four potential targets of 10 active anti-AD ingredients of GF were identified, among which genkwanin (GK) had the greatest number of AD-related targets. KEGG pathway enrichment analysis showed that the most significantly enriched signaling pathway of GF against AD was Alzheimer disease (hsa05010). More importantly, 29 of the 84 targets were significantly correlated with tau, Aβ or both Aβ and tau pathology. In addition, GO analysis suggested that the main biological processes of GF in AD treatment were the regulation of chemical synaptic transmission (GO:0007268), neuron death (GO:0070997), amyloid-beta metabolic process (GO:0050435), etc. We further investigated the anti-AD effects of GK using N2A-APP cells (a classical cellular model of AD). Treatment N2A-APP cells with 100 μM GK for 48 h affected core targets related to tau pathology (such as CDK5 and GSK3β). In conclusion, these findings indicate that GF exerts its therapeutic effects on AD by acting directly on multiple pathological processes of AD.

## INTRODUCTION

Alzheimer’s disease (AD) is a complex and progressive neurodegenerative disorder that is considered the most common type of dementia [1]. In China, it was reported that 3.2% of people aged ≥ 60 years old were AD patients in 2018 [2]. With the rapid increase in the aging population, the yearly prevalence of AD has been predicted to increase to 5.35% in 2021 [2]. The etiology of AD has not been fully deciphered. Currently, the typical events in the pathogenesis of AD are extracellular amyloid-β (Aβ) plaques and aberrant hyperphosphorylated tau protein [3, 4]. Most studies considered Aβ to be an upstream regulator of tau [5, 6] in AD pathogenesis that triggers an abnormal increase in postsynaptic Ca^2+^ flux [7] and synaptic/neurotransmission dysfunction [8], leading to apoptotic neuronal death [9]. Current treatments of AD primarily include cholinesterase inhibitors (tacrine [10], donepezil [11], rivastigmine [11], and galantamine [11]) and the noncompetitive N-methyl-D-aspartic acid (NMDA) receptor inhibitor memantine [12]. These medicines can ameliorate the symptoms of AD but have a limited effect on delaying the onset of dementia. It is worth noting that the Food and Drug Administration recently approved Aduhelm (aducanumab) for the treatment of AD. Over the last decade, disease-modifying drugs for AD treatment have been lacking [13]. The effects of AD therapies are limited due to the unelucidated pathological mechanisms of AD and the side effects of drugs, such as hepatotoxicity, dizziness, and headache [14]. Therefore, drugs active against one or more of these triggers could be a promising strategy.

Traditional Chinese medicines have been widely used to treat cognitive impairment and AD [15, 16]. *Ginkgo Folium* (GF) extract and its components have long been reported to have extensive effects, such as neuroprotective, cardioprotective and anticancer effects [17, 18]. Text mining results also indicated the GF is one of the top 10 anti-AD herbs [16]. Some studies have suggested that GF can protect neurons against glutamate neurotoxicity by reducing the elevation of Ca^2+^ [19, 20]. Other studies suggested that GF may inhibit the formation of Aβ peptide fibrils [21, 22]. A recent study showed a beneficial effect of GF on hippocampal neurogenesis [23]. Moreover, GF is generally regarded as safe, without excessive side effects [18]. Numerous clinical trials have shown that the GF extract EGb761 can improve cognitive function and ameliorate symptoms in patients with AD [24, 25]. In previous studies, EGb761 showed potential benefits in multiple AD animal models by regulating inflammation, exerting antioxidative effects and decreasing tau hyperphosphorylation [26, 27]. Additionally, GF appears to be a promising plant-based dietary supplement with therapeutic benefits. However, the therapeutic mechanisms of action of GF in AD remain unclear, and the underlying anti-AD mechanism of GF must be elucidated.

**Figure 1:**
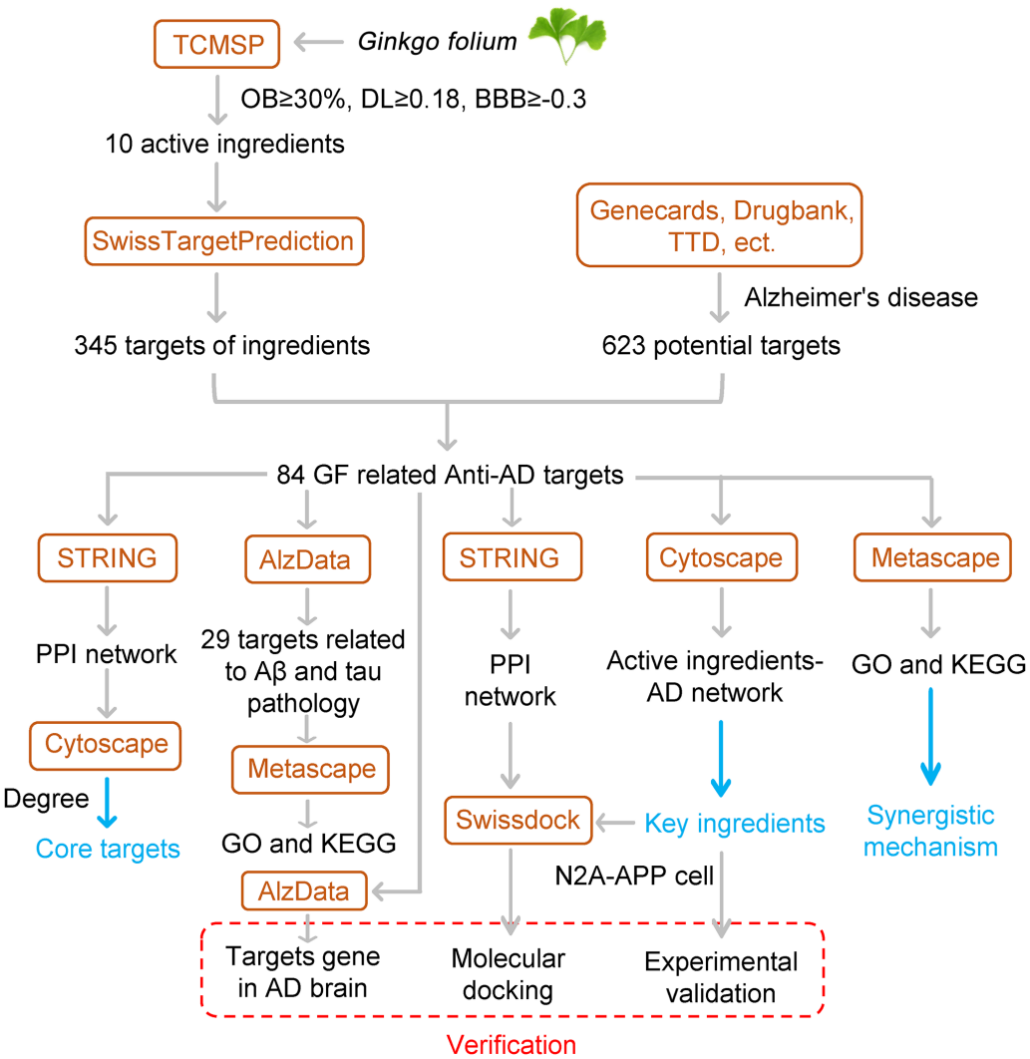
Flowchart of the study.

As an important part of systematic biology, network pharmacology has been widely used in drug discovery and development [28]. To date, this application has been successfully used to illustrate the multitarget effects of traditional Chinese medicines in several diseases [29–31]. This study aimed to investigate the main ingredient of GF and its anti-AD mechanisms through network pharmacology approach and experimental verification. Our protocol is shown as (Figure 1). Noteworthy, we screened out 29 targets correlated with Aβ and tau pathology from an AD database. Furthermore, we also used the human high-throughput omics data and molecular docking to validate the targets of GF against AD. Our findings provide a systemic pharmacology basis for the anti-AD effects of GF.

## RESULTS

### Active anti-AD ingredients and target proteins of GF

**Figure 2:**
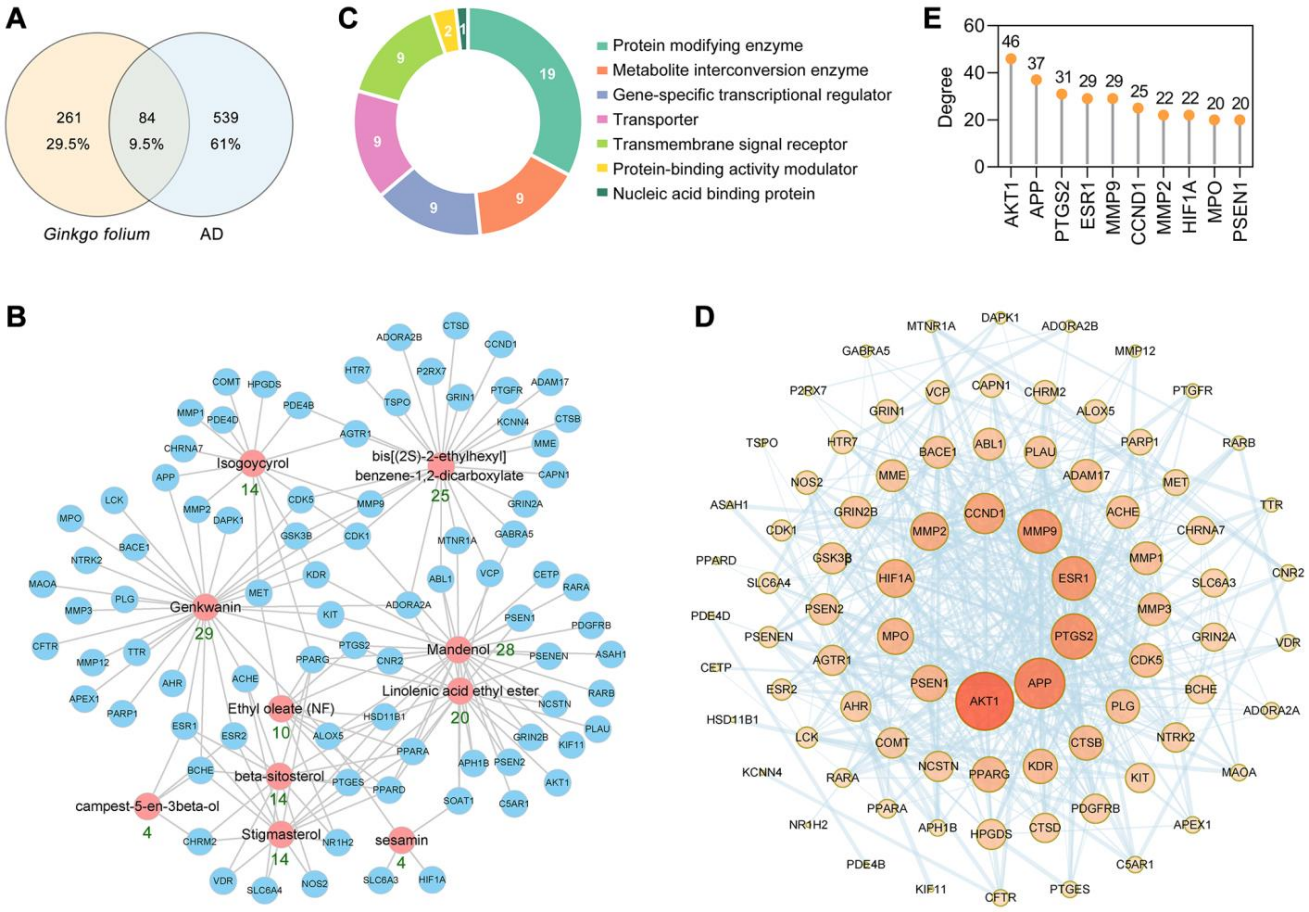
**PPI network construction for the target proteins of GF against AD.** (**A**) The intersection of GF and AD targets. (**B**) The main active ingredients-AD target network diagram of GF against AD. The active ingredients nodes are colored in red, and blue nodes represent target proteins. (**C**) Panther classification categorized target proteins of GF against AD. (**D**) PPI network of GF against AD. Nodes, targets; edges, interaction among targets. The darker the color and the larger the node, the higher the degree. The thickness of the edges represents the combined score. (**E**) The top 10 core targets were excavated according to the degree. The numbers above the dots represent degree.

The Traditional Chinese Medicine Systems Pharmacology Database and Analysis Platform (TCMSP) database [32] is the largest pharmacological data platforms for traditional Chinese medicines. A total of 10 active ingredients of GF were retrieved from the TCMSP database according to the following screening criteria: oral bioavailability (OB) ≥ 30%, drug likeness (DL) score ≥ 0.18 and a blood-brain barrier (BBB) score ≥ –0.3 (Table 1). These 10 ingredients were also analyzed for their suitability for the use as a drug, based on Lipinski's Rule of Five (Table 1). The SwissTargetPrediction database [33] was used to obtain the potential targets of GF based on their structures. In total, 345 potential target proteins were predicted. To identify the AD-associated targets, 623 targets were collected from the GeneCards database, DrugBank database, Therapeutic Target Database (TTD) [34] and Chemogenomics Database for Alzheimer's disease. Ultimately, the Venn diagram identified 84 targets associated with both GF and AD for further analysis (Figure 2A). Detailed information about these common targets is shown in Table 2. The 10 active ingredient-AD target network for the activity of GF against AD is shown in Figure 2B. Among the identified interactions, the active ingredient genkwanin (GK) had 29 AD-related therapeutic targets and fulfill Lipinski’s rule of five. Among these targets, APP, ESR1, MMP2, MMP9, MPO and PTGS2 were core targets. Twenty-nine herbs containing GK including GF (Supplementary Table 1).

**Table 1:** The main active ingredients in GF.

**Name**	**Formula**	**MW (g/mol)**	**Hdon**	**Hacc**	**Rbon**	**LogP**	**OB (%)**	**BBB**	**DL**
Beta-sitosterol	C_29_H_50_O	414.79	1	1	6	7.19	36.91	0.99	0.75
Stigmasterol	C_29_H_48_O	412.77	1	1	5	6.96	43.83	1	0.76
Bis[(2S)-2-ethylhexyl] benzene-1,2-dicarboxylate	C_24_H_38_O_4_	390.62	0	4	16	6.17	43.59	0.68	0.35
Mandenol	C_20_H_36_O_2_	308.56	0	2	16	6.09	42	1.14	0.19
Sesamin	C_20_H_18_O_6_	354.38	0	6	2	2.79	56.55	–0.08	0.83
Ethyl oleate (NF)	C_20_H_38_O_2_	310.58	0	2	17	6.33	32.4	1.1	0.19
Campest-5-en-3beta-ol	C_28_H_48_O	400.76	1	1	5	6.9	37.58	0.94	0.71
Genkwanin	C_16_H_12_O_5_	284.28	2	5	2	2.5	37.13	–0.24	0.24
Linolenic acid ethyl ester	C_20_H_34_O_2_	306.54	0	2	15	5.82	46.1	1.09	0.2
Isogoycyrol	C_21_H_18_O_6_	366.39	1	6	1	3.79	40.36	0	0.83

**Table 2:** Target information of GF against AD.

**Number**	**Gene ID**	**Protein description**	**Gene symbol**	**Number**	**Gene ID**	**Protein description**	**Gene symbol**
1	25	ABL proto-oncogene 1, non-receptor tyrosine kinase	ABL1	43	3832	kinesin family member 11	KIF11
2	43	acetylcholinesterase (Cartwright blood group)	ACHE	44	3815	KIT proto-oncogene, receptor tyrosine kinase	KIT
3	6868	ADAM metallopeptidase domain 17	ADAM17	45	3932	LCK proto-oncogene, Src family tyrosine kinase	LCK
4	135	adenosine A2a receptor	ADORA2A	46	4128	monoamine oxidase A	MAOA
5	136	adenosine A2b receptor	ADORA2B	47	4233	MET proto-oncogene, receptor tyrosine kinase	MET
6	185	angiotensin II receptor type 1	AGTR1	48	4311	membrane metalloendopeptidase	MME
7	196	aryl hydrocarbon receptor	AHR	49	4312	matrix metallopeptidase 1	MMP1
8	207	AKT serine/threonine kinase 1	AKT1	50	4321	matrix metallopeptidase 12	MMP12
9	240	arachidonate 5-lipoxygenase	ALOX5	51	4313	matrix metallopeptidase 2	MMP2
10	328	apurinic/apyrimidinic endodeoxyribonuclease 1	APEX1	52	4314	matrix metallopeptidase 3	MMP3
11	83464	aph-1 homolog B, gamma-secretase subunit	APH1B	53	4318	matrix metallopeptidase 9	MMP9
12	351	amyloid beta precursor protein	APP	54	4353	myeloperoxidase	MPO
13	427	N-acylsphingosine amidohydrolase 1	ASAH1	55	4543	melatonin receptor 1A	MTNR1A
14	23621	beta-secretase 1	BACE1	56	23385	nicastrin	NCSTN
15	590	butyrylcholinesterase	BCHE	57	4843	nitric oxide synthase 2	NOS2
16	728	complement C5a receptor 1	C5AR1	58	7376	nuclear receptor subfamily 1 group H member 2	NR1H2
17	823	calpain 1	CAPN1	59	4915	neurotrophic receptor tyrosine kinase 2	NTRK2
18	595	cyclin D1	CCND1	60	5027	purinergic receptor P2X 7	P2RX7
19	983	cyclin dependent kinase 1	CDK1	61	142	poly(ADP-ribose) polymerase 1	PARP1
20	1020	cyclin dependent kinase 5	CDK5	62	5142	phosphodiesterase 4B	PDE4B
21	1071	cholesteryl ester transfer protein	CETP	63	5144	phosphodiesterase 4D	PDE4D
22	1080	CF transmembrane conductance regulator	CFTR	64	5159	platelet derived growth factor receptor beta	PDGFRB
23	1129	cholinergic receptor muscarinic 2	CHRM2	65	5328	plasminogen activator, urokinase	PLAU
24	1139	cholinergic receptor nicotinic alpha 7 subunit	CHRNA7	66	5340	plasminogen	PLG
25	1269	cannabinoid receptor 2	CNR2	67	5465	peroxisome proliferator activated receptor alpha	PPARA
26	1312	catechol-O-methyltransferase	COMT	68	5467	peroxisome proliferator activated receptor delta	PPARD
27	1508	cathepsin B	CTSB	69	5468	peroxisome proliferator activated receptor gamma	PPARG
28	1509	cathepsin D	CTSD	70	5663	presenilin 1	PSEN1
29	1612	death associated protein kinase 1	DAPK1	71	5664	presenilin 2	PSEN2
30	2099	estrogen receptor 1	ESR1	72	55851	presenilin enhancer, gamma-secretase subunit	PSENEN
31	2100	estrogen receptor 2	ESR2	73	9536	prostaglandin E synthase	PTGES
32	2558	gamma-aminobutyric acid type A receptor subunit alpha5	GABRA5	74	5737	prostaglandin F receptor	PTGFR
33	2902	glutamate ionotropic receptor NMDA type subunit 1	GRIN1	75	5743	prostaglandin-endoperoxide synthase 2	PTGS2
34	2903	glutamate ionotropic receptor NMDA type subunit 2A	GRIN2A	76	5914	retinoic acid receptor alpha	RARA
35	2904	glutamate ionotropic receptor NMDA type subunit 2B	GRIN2B	77	5915	retinoic acid receptor beta	RARB
36	2932	glycogen synthase kinase 3 beta	GSK3B	78	6531	solute carrier family 6 member 3	SLC6A3
37	3091	hypoxia inducible factor 1 subunit alpha	HIF1A	79	6532	solute carrier family 6 member 4	SLC6A4
38	27306	hematopoietic prostaglandin D synthase	HPGDS	80	6646	sterol O-acyltransferase 1	SOAT1
39	3290	hydroxysteroid 11-beta dehydrogenase 1	HSD11B1	81	706	translocator protein	TSPO
40	3363	5-hydroxytryptamine receptor 7	HTR7	82	7276	transthyretin	TTR
41	3783	potassium calcium-activated channel subfamily N member 4	KCNN4	83	7415	valosin containing protein	VCP
42	3791	kinase insert domain receptor	KDR	84	7421	vitamin D receptor	VDR

Furthermore, the 84 anti-AD target proteins of GF were categorized into 7 different classes based on their cellular function, and the protein-modifying enzyme (PC00260, 32.8%) class contained the greatest number of these proteins (Figure 2C). Among the protein-modifying enzymes, AKT1, CDK1, CDK5, DAPK1 and glycogen synthase kinase 3β (GSK3β) are non-receptor serine/threonine protein kinases; MME, MMP12, MMP1, MMP2, MMP3 and MMP9 are metalloproteases; and PLAU and PLG are serine proteases.

The protein-protein interaction (PPI) network of the 84 AD-associated GF targets was constructed using the STRING database version 11.0 [35]. The PPI network contained 84 nodes and 469 edges, and the average node degree was 11.2 (Figure 2D). AKT1, APP, PTGS2, ESR1, MMP9, CCND1, MMP2, HIF1A, MPO and PSEN1 were identified as the core targets ranked by degree (Figure 2E). The above results indicated that GF could protect against AD through multiple targets and biological functions.

### Gene ontology (GO) biological process and Kyoto Encyclopedia of Genes and Genomes (KEGG) pathway enrichment analyses

**Figure 3:**
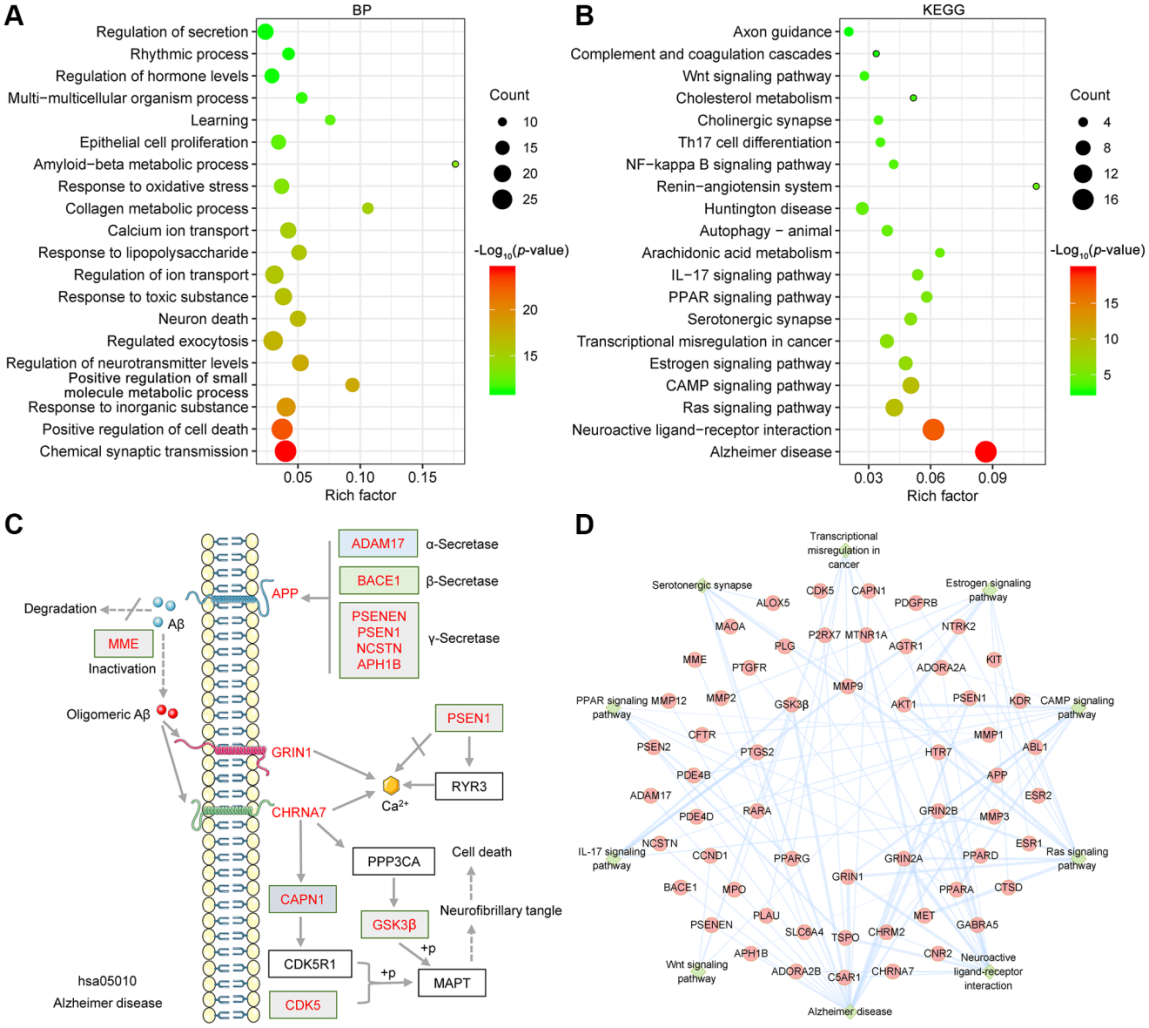
**Bioinformatics analysis of target proteins of GF against AD.** (**A**) Top 20 bubble chart of biological process of GO enrichment analysis. (**B**) The top 20 KEGG pathways are presented in the bubble chart. (**C**) The genes involved in the Alzheimer disease pathway are presented in the mechanistic diagram of AD pathology. (**D**) Top 10 significantly enriched KEGG pathways are shown as a network diagram. Red circle nodes represent target proteins, and green diamond nodes represent enriched KEGG pathways. X-axis, rich factor; bubble size, the number of targets enriched; bubble color, *p* value.

To investigate the potential synergistic mechanism of the GF against AD, GO and KEGG enrichment analyses were performed by the Metascape database [36]. The main GO biological processes identified were chemical synaptic transmission (GO:0007268), positive regulation of cell death (GO:0010942), response to inorganic substance (GO:0010035), positive regulation of small molecule metabolic process (GO:0062013), regulation of neurotransmitter levels (GO:0001505), regulated exocytosis (GO:0045055), neuron death (GO:0070997) and so on (Figure 3A). The primary KEGG pathways identified were Alzheimer disease (hsa05010), neuroactive ligand-receptor interaction (ko04080), estrogen signaling pathway (hsa04915), Ras signaling pathway (hsa04014), cAMP signaling pathway (ko04024), Wnt signaling pathway (ko04310), serotonergic synapse (hsa04726) and so on (Figure 3B). Notably, Alzheimer disease (hsa05010) and neuroactive ligand-receptor interaction (ko04080) pathway exhibited the greatest number of target connections (degree = 17). Detailed data from the KEGG pathway enrichment analysis is shown in Table 3. The targets involved in the Alzheimer disease pathway are presented in the mechanistic diagram of AD pathology. The identified targets shown in red were involved in two major pathological processes of AD (Figure 3C). Moreover, the top 10 pathways in the KEGG pathway-target network are shown in Figure 3D.

**Table 3:** KEGG pathway enrichment analysis of GF against AD.

**Pathway**	**Rich factor**	***p* value**	**Count**	**Symbols**
Alzheimer disease	0.09	1.95E-20	17	APP, CAPN1, CDK5, GRIN1, GRIN2A, GRIN2B, GSK3β, MME, MMP12, PSEN1, PSEN2, PTGS2, ADAM17, NCSTN, BACE1, PSENEN, APH1B
Neuroactive ligand-receptor interaction	0.06	7.2E-18	17	ADORA2A, ADORA2B, AGTR1, TSPO, C5AR1, CHRM2, CHRNA7, CNR2, GABRA5, GRIN1, GRIN2A, GRIN2B, HTR7, MTNR1A, P2RX7, PLG, PTGFR
Estrogen signaling pathway	0.05	2.94E-07	7	AKT1, CTSD, ESR1, ESR2, MMP2, MMP9, RARA
Ras signaling pathway	0.04	3.52E-10	11	ABL1, AKT1, GRIN1, GRIN2A, GRIN2B, HTR7, KDR, KIT, MET, NTRK2, PDGFRB
CAMP signaling pathway	0.05	4.37E-10	10	ADORA2A, AKT1, CFTR, CHRM2, GRIN1, GRIN2A, GRIN2B, PDE4B, PDE4D, PPARA
Wnt signaling pathway	0.03	0.000918	4	CCND1, GSK3β, PPARD, PSEN1
Transcriptional misregulation in cancer	0.04	1.21E-06	7	MET, MMP3, MMP9, MPO, PLAU, PPARG, RARA
Serotonergic synapse	0.05	1.61E-06	6	ALOX5, APP, HTR7, MAOA, PTGS2, SLC6A4
PPAR signaling pathway	0.06	6.21E-06	5	TSPO, MMP1, PPARA, PPARD, PPARG
IL-17 signaling pathway	0.05	9.11E-06	5	GSK3β, MMP1, MMP3, MMP9, PTGS2
Arachidonic acid metabolism	0.06	3.69E-05	4	ALOX5, PTGS2, PTGES, HPGDS
Autophagy - animal	0.04	4.27E-05	5	AKT1, CTSB, CTSD, DAPK1, HIF1A
Huntington disease	0.03	5.64E-05	6	APEX1, GRIN1, GRIN2B, PPARG, PTGS2, SLC6A4
Renin-angiotensin system	0.11	7.24E-05	3	AGTR1, MME, MMP12
NF-kappa B signaling pathway	0.04	0.000195	4	PARP1, LCK, PLAU, PTGS2
Th17 cell differentiation	0.04	0.000366	4	AHR, HIF1A, LCK, RARA
Cholinergic synapse	0.03	0.000405	4	ACHE, AKT1, CHRM2, CHRNA7
Cholesterol metabolism	0.05	0.000714	3	TSPO, CETP, SOAT1
Complement and coagulation cascades	0.03	0.002458	3	C5AR1, PLAU, PLG
Axon guidance	0.02	0.002906	4	ABL1, CDK5, GSK3β, MET

### Bioinformatic analysis of target proteins correlated with Aβ and tau pathology

**Figure 4:**
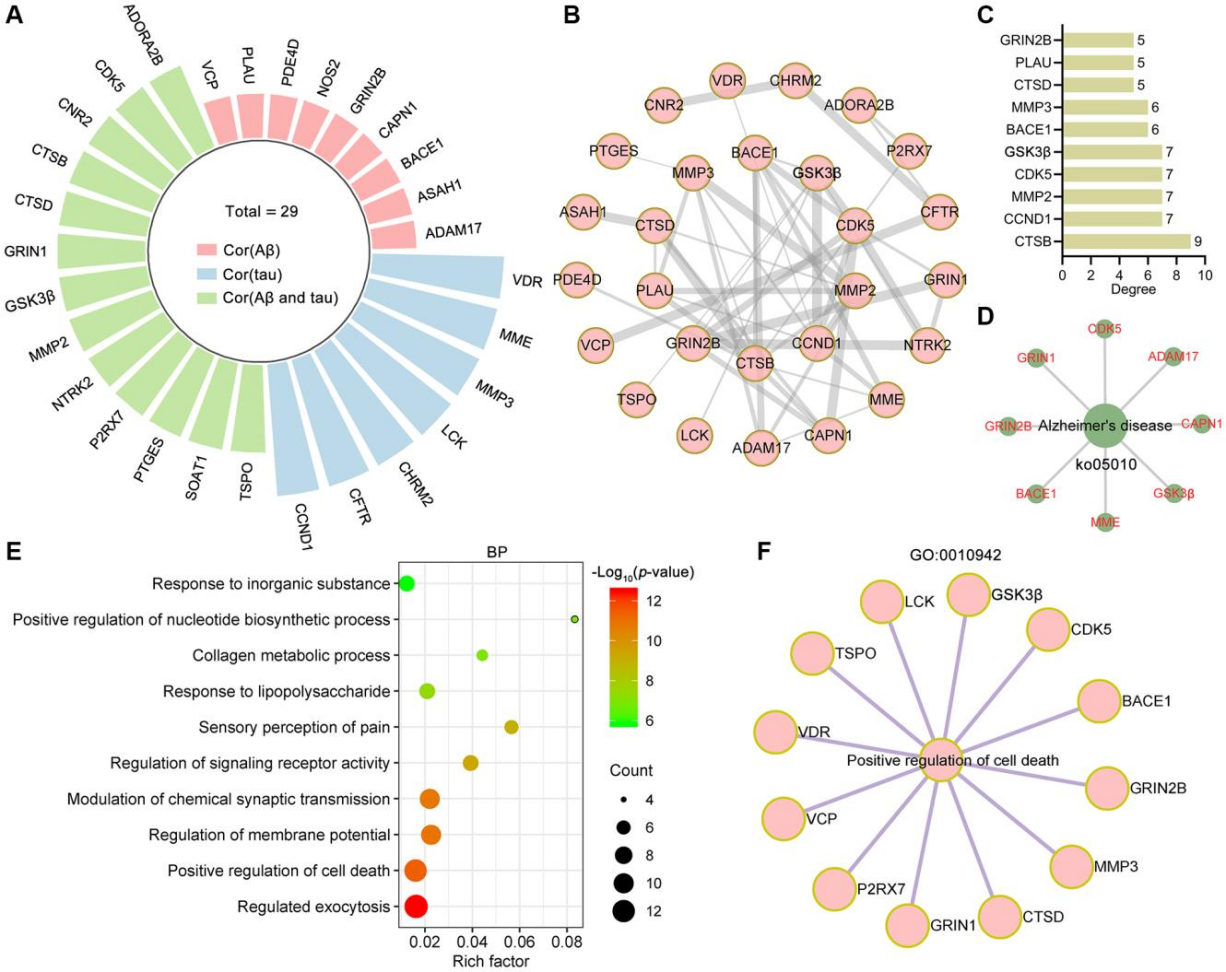
**Bioinformatics analysis of targets related to Aβ and tau pathology.** (**A**) Radial bar plot showing the target proteins significantly correlated with tau, Aβ or Aβ and tau. (**B**) PPI network construction for proteins correlated with tau, Aβ or Aβ and tau. (**C**) The top 10 core targets from the PPI network were ranked by degree. (**D**) Network diagram showing target proteins involved in Alzheimer's disease (ko05010). (**E**) Top 10 bubble chart of biological process of GO enrichment analysis. X-axis, rich factor; bubble size, the number of targets enriched; bubble color, *p* value. (**F**) Network diagram showing target proteins involved in positive regulation of cell death (GO: 0010942).

To analyze the targets related to Aβ and tau pathology, the AlzData database [37] was used. Twenty-nine of the 84 targets were significantly correlated with tau, Aβ or both Aβ and tau (Figure 4A). Among these 29 targets, 27 targets formed a complex PPI network containing 27 nodes and 50 edges with an average node degree of 3.7 (Figure 4B). CTSB, CCND1, MMP2, CDK5, GSK3β, BACE1, MMP3, CTSD, PLAU and GRIN2β were identified as core targets (Figure 4C). The network diagram showing the target proteins involved in the Alzheimer disease pathway (ko05010) is shown in Figure 4D. In the GO biological process analysis, these targets were enriched mainly in regulated exocytosis (GO:0045055), positive regulation of cell death (GO:0010942), regulation of membrane potential (GO:0042391), modulation of chemical synaptic transmission (GO:0050804), regulation of signaling receptor activity (GO:0010469) and so on (Figure 4E). Twelve targets involved in the positive regulation of cell death process were identified (Figure 4F).

### Gene Expression Omnibus (GEO) dataset analysis of AD-associated GF targets related to Aβ and tau pathology

**Figure 5:**
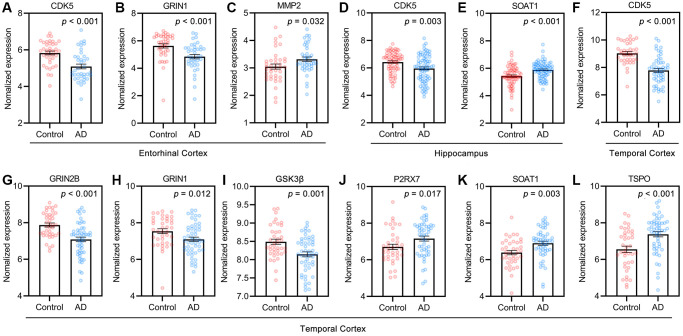
**Targets of GF against AD in control and AD groups of GEO dataset.** (**A**–**C**) Entorhinal cortex, *n* = 39 in each group. (**D**, **E**) Hippocampus, *n* = 66 in healthy control, *n* = 74 in the AD patients. (**F**–**L**) Temporal cortex, *n* = 39 in healthy control, *n* = 52 in the AD patients. Values are presented as mean ± standard errors mean (SEM).

The normalized expression values of the targets related to AD pathology in the healthy control and AD groups in the GEO dataset were analyzed with the “Differential expression” module of the AlzData database (Figure 5). Among these targets, CDK5 and GRIN1 were significantly downregulated (Figure 5A, 5B), and MMP2 was significantly upregulated in the entorhinal cortex in AD patients compared to controls (Figure 5C). The expression of CDK5 (Figure 5D) was significantly downregulated but the expression of SOAT1 (Figure 5E) was upregulated in the hippocampus in AD patients compared to controls. The expression of CDK5 (Figure 5F), GRIN2β (Figure 5G), GRIN1 (Figure 5H) and GSK3β (Figure 5I) was significantly downregulated but the expression of P2RX7 (Figure 5J), SOAT1 (Figure 5K) and TSPO (Figure 5L) was upregulated in the temporal cortex in AD patients compared to controls. These results suggested that the potential pivotal role of GF targets in AD pathogenesis.

### Receiver operating characteristic (ROC) analysis of targets related to Aβ and tau pathology

**Figure 6:**
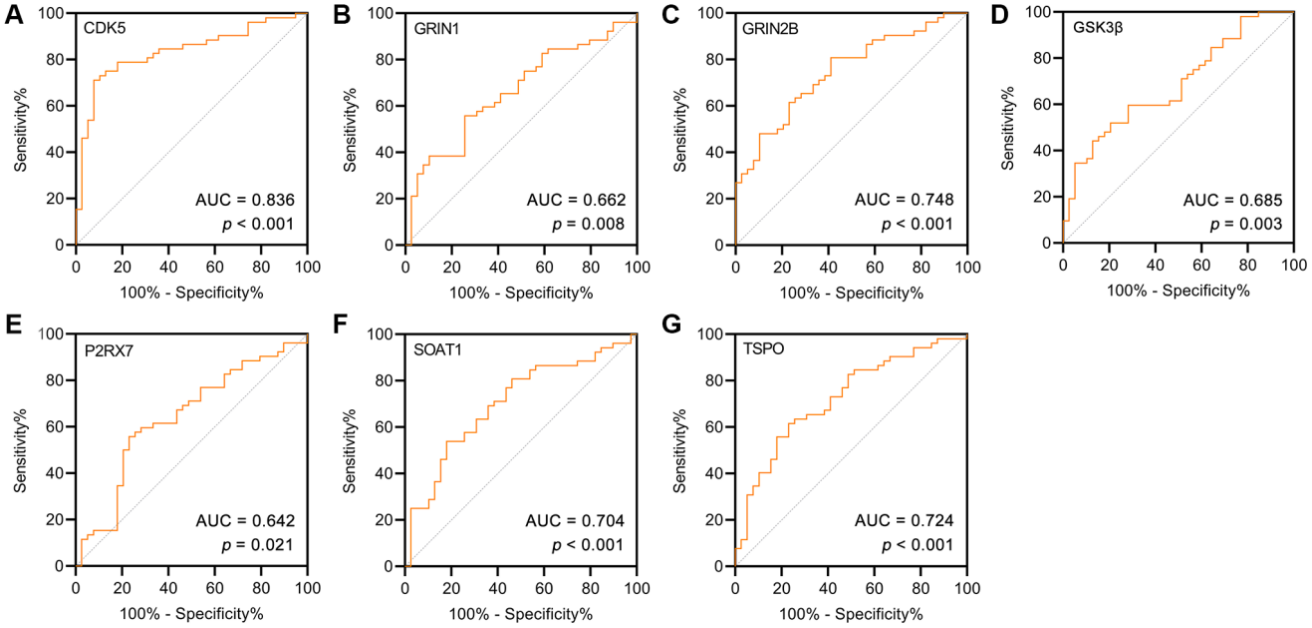
(**A**–**G**) ROC curve for the AD and controls on potential targets of GF. As a reference, a curve with an AUC of 0.5 was plotted (dashed line).

ROC curve analysis was used to describe the discrimination accuracy of these targets in the diagnosis of AD. The area under the ROC curve (AUC) is a combined measure of sensitivity and specificity [38]. The closer that the AUC is to 1, the better that the diagnostic performance of the test is. The practical lower limit of the AUC for a diagnostic test is 0.5. The AUC values indicated that CDK5, GRIN1, GRIN2β, GSK3β, P2RX7, SOAT1 and TSPO had high diagnostic performance for AD (Figure 6A–6G). Moreover, CDK5 had the best diagnostic performance, followed by GRIN2β.

### Molecular docking of GK with Aβ and tau pathology related targets

To validate the binding of GK to targets related to Aβ and tau pathology, the molecular docking procedure was performed using LeDock [39]. The lower the score, the better the docking effect. Molecular docking results of GK to targets were shown in Table 4. Among these targets, GK showed the highest binding energy to MMP3, LCK, CDK5, NTRK2, GSK3β, BACE1 and CFTR, and the scores were –6.56, –6.5, –6.41, –6.01, –5.86, –5.61 and –5.61 kcal/mol, respectively.

**Table 4:** Molecular docking of GK with Aβ and tau pathology associated GK targets.

**Target**	**PDB**	**Score (kcal/mol)**
MMP3	3OHO	–6.56
LCK	3KMM	–6.5
CDK5	4AU8	–6.41
NTRK2	4AT3	–6.01
GSK3β	2O5K	–5.86
BACE1	3QBH	–5.61
CFTR	3GD7	–5.61

**Figure 7:**
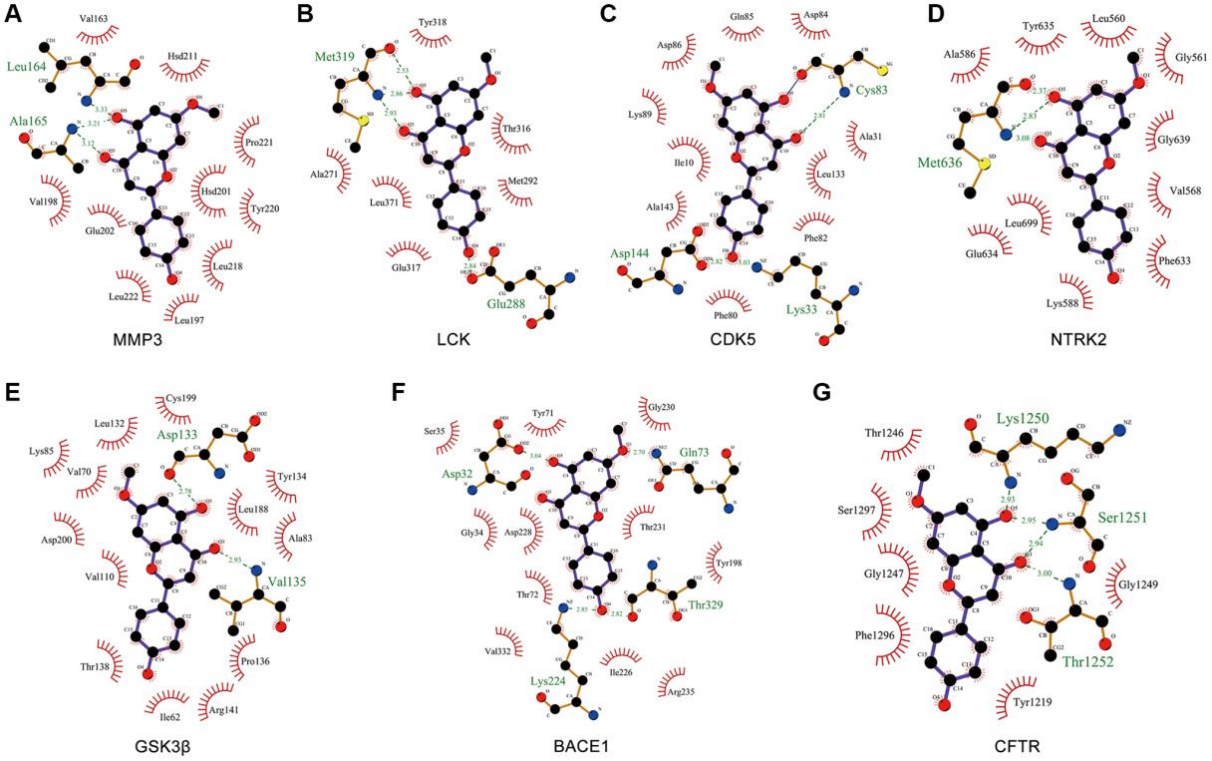
**Molecular docking of GK targets related to Aβ and tau pathology with GK.** (**A**–**G**) The LigPlus schematic 2D representation of GK-targets interactions. Hydrogen bonds between GK and targets are represented by green dashed lines. The amino acid residues of targets interacted with GK are shown as brown sticks and labeled in green.

Ligand-protein interactions were calculated using LigPlot [40]. Figure 7 demonstrated that GK binds tightly in the MMP3, LCK, CDK5, NTRK2, GSK3β, BACE1 and CFTR binding pocket and stabilized by hydrogen bonds interactions. Specifically, GK formed potential interactions with residues Leu164 and Ala165 of MMP3 through hydrogen bonds (Figure 7A). The distance between GK and Leu164 was 3.33 Å, the distance of two hydrogen bonds between GK and Ala165 were 3.21 and 3.12 Å respectively (Figure 7A). Moreover, GK formed potential interactions with residues Met319 and Glu288 of LCK through hydrogen bonds (Figure 7B). The distance of three hydrogen bonds between GK and Met319 were 2.53, 2.86 and 2.93 Å respectively, the distance between GK and Glu288 was 2.84 Å (Figure 7B). GK bound with CDK5 by forming three hydrogen bonds at Asp144, Cys83 and Lys33 residues (Figure 7C). GK bound with NTRK2 by forming three hydrogen bonds at Met636 residues (Figure 7D). GK formed potential interactions with residues Asp133 and Val135 of GSK3β through hydrogen bonds (Figure 7E). GK bound with BACE1 by forming four hydrogen bonds at Asp32, Lys224, Gln73 and Thr329 residues (Figure 7F). In addition, GK also formed potential interactions with residues Lys1250, Ser1251 and Thr1252 of CFTR through hydrogen bonds (Figure 7G). These findings suggested that GK has significant binding to Aβ and tau pathology related targets.

### The effect of GK on potential targets in N2A-APP cells

**Figure 8:**
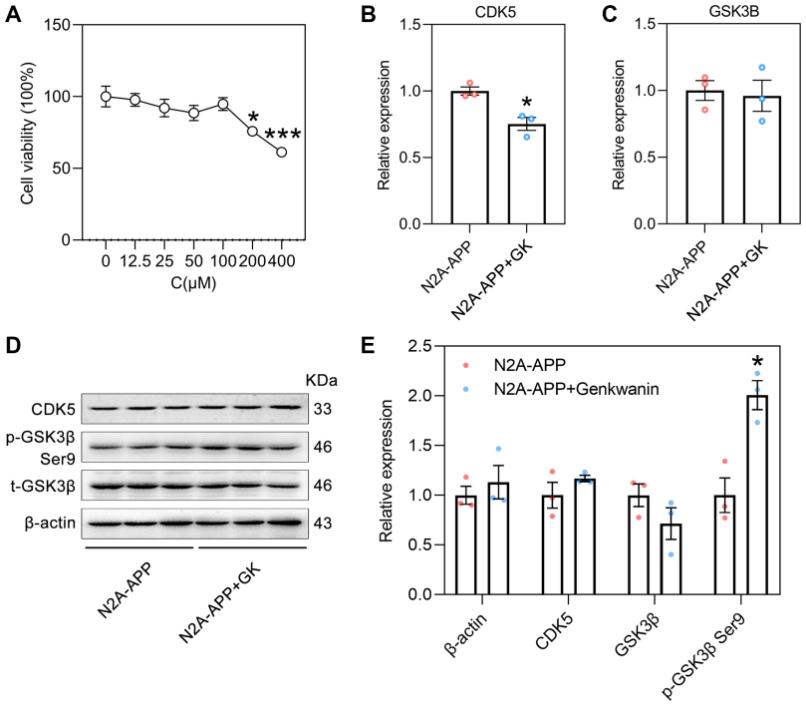
**The effect of GK on CDK5 and GSK3β in N2A-APP cells.** (**A**) The viability of genkwanin treated N2A-APP cells were measured at different concentration using CCK-8 analysis for 48 h (*n* = 5). Data were expressed as the means ± SEM. ^*^p < 0.05, ^***^p < 0.001 vs 0 μM. (**B**, **C**) The level of CDK5 and GSK3β were normalized to the level of β-actin mRNA (*n* = 3/group). (**D**, **E**) Protein level of CDK5, phosphorylated GSK3β (p-GSK3β, Ser9) and total GSK3β (t-GSK3β) were measured by Western blotting and quantitatively analyzed (*n* = 3/group). β-actin was used as a protein loading control. N2A-APP cells treated with 0.1% DMSO solvent served as controls. Data were expressed as the means ± SEM. ^*^p < 0.05, ^**^p < 0.01 vs N2A-APP group.

N2A-APP cells, a classical cellular model of AD, are a stable mouse neuroblastoma cell line (Neuro-2A) overexpressing Swedish mutant APP695 [41, 42]. This cell line can be used to verify the pathological processes of Aβ generation and tau hyperphosphorylation [43]. The main active ingredient-AD target network for the activity of GF against AD showed that GK had the highest degree of connectivity (Figure 2B). Therefore, this study used N2A-APP cells to examine the anti-AD effect of GK. N2A-APP cells were treated with different concentrations of GK for 48 h. The cell counting kit-8 (CCK-8) assay showed that the viability of cells treated with 200 μM (75.87 ± 1.25) or 400 μM (61.32 ± 0.93) GK was reduced. The CCK-8 assay showed that the optimal concentration of GK to treat N2A-APP cells was 100 μM (94.81 ± 1.96) (Figure 8A). The RT-PCR results showed that the mRNA level of CDK5 (0.75 ± 0.03) was decreased by ~25% (Figure 8B); however, the mRNA level of GSK3β (0.96 ± 0.07) was not changed in the N2A-APP+GK group compared with N2A-APP group (treated with 0.1% DMSO solvent served as controls) (Figure 8C). Western blot analysis showed that the protein level of p-GSK3β (2 ± 0.08) were increased twofold by GK, and the protein level of CDK5 and *t*-GSK3β was not changed in the N2A-APP+GK group (Figure 8D–8E).

## DISCUSSION

To investigate the benefits of GF on AD and uncover the potential molecular mechanisms, a network pharmacology approach was used in this study. Eighty-four potential targets of 10 anti-AD ingredients in GF were identified using network pharmacology strategies. Among the 10 active compounds, GK had the greatest number (29) of anti-AD targets and 6 core targets are involved. In addition to meeting the screening criteria for active ingredients (OB ≥ 30%, DL score ≥ 0.18 and BBB score ≥ –0.3), GK also fulfill Lipinski’s rule of five. This revealed that the GK has the potential to be a promising therapeutic drug. Previous studies have shown that GK has a variety of pharmacological effects such as antibacterial [44], anti-inflammatory [45], antiplasmodial [46], antitumor [47], radical scavenging [48] and chemopreventive [49] effects. GK can inhibit proinflammatory signaling through regulation of the miR-101/MKP-1/MAPK pathway in active macrophages [45]. A recent study showed that GK protected neurons in an animal model of Parkinson’s disease [50]. Accordingly, we identified GK as the key ingredient and selected GK for subsequent experiments.

Apart from the GK, β-Sitosterol (10 mg/kg/day for 5 days) improved working memory and motor coordination in transgenic animals [51]. Stigmasterol might be beneficial in preventing AD through regulation of APP processing [52]. Sesamin can potentially protect neuronal cells (SH-SY5Y) against oxidative stress via the SIRT1-SIRT3-FOXO3a signaling pathway [53]. A previous network analysis showed that β-sitosterol and stigmasterol target the PI3K/AKT pathway to combat AD-associated pathobiology [54]. In addition, the neuroprotective effect of sesamin mediated via AKT1 was verified in a BV-2 cell model of ischemia/hypoxia [55]. The results of the present study demonstrated that AKT1 was also the core anti-AD target of stigmasterol, β-sitosterol and sesamin. Deficits in ACHE and BCHE are thought to be associated with the initiation and development of AD [56], and β-sitosterol was reported to mediate memory deficits as an AChE and BChE inhibitor in AD transgenic animals [51].

GF could exert its anti-AD effect through multi-targets and multi-pathways. Core targets play a crucial role in the entire network and may play a vital role in the GF therapy. AKT1 is a vital prosurvival kinase that suppresses apoptotic signaling. Increased AKT1 phosphorylation reduced the level of Aβ and subsequently improved cognitive ability in a rat model of AD [5]. Acetylcholinesterase (AChE) and butyrylcholinesterase (BChE) are important targets for the development of anti-AD drugs. In the present study, AChE and BChE were identified as AD-related targets of GF, suggesting that GF can improve symptoms or slow the progression of AD. We further analyzed correlations of GF targets with the AD pathology (Aβ and tau). Twenty-nine targets of GF were involved in AD pathology; among these targets, 7 were associated with tau, 9 were associated with Aβ, and the remaining 13 were related to both. These targets were significantly enriched in several biological processes, for example, chemical synaptic transmission, positive regulation of cell death and response to inorganic substance. These three biological processes mediate AD pathogenesis [57–59]. In addition, the targets were significantly enriched in the Alzheimer disease KEGG pathway, implying that GF exerts its therapeutic effects on AD by acting directly on multiple pathological processes of AD.

The anti-AD effect of GF involved in multiple biological processes, such as regulation of exocytosis, cell death and chemical synaptic transmission. GF was confirmed to facilitate glutamate exocytosis from rat hippocampal nerve terminals to improve memory and cognitive function in AD patients [60]. CDK5, GSK3β and BACE1 are involved in regulating cell death. Neuronal death, an important aspect of AD pathogenesis [61], was reversed by GF [62]. Decreased synaptic density, compromised chemical synaptic transmission and defective synaptic plasticity are hallmark synaptic pathologies accompanying AD [57], and CDK5, GSK3β and BACE1 are essential for maintaining synaptic functions [63–65]. Acute application of EGb761 (a standardized extract of GF) significantly increased synaptic plasticity and excitability in aged C57BL/6 mice [66]. The results of the present study demonstrated that CDK5, GSK3β and BACE1 are involved in the dysfunctional biological processes of AD, which can be inhibited by GF treatment. These results further illustrated that GF exerts beneficial effects on AD through multiple biological processes, consistent with the findings of previous studies.

Aggregation of Aβ is the typical pathological feature of AD. KEGG pathway enrichment analysis showed that the target proteins were enriched mainly in the Alzheimer disease pathway; specifically, 17 target proteins were enriched in this pathway. ADAM17, BACE1, PSENEN, PSEN1, NCSTN and APH1B regulate the production of Aβ by affecting APP splicing enzymes [67–69]. MME is a principal peptidase involved in the degradation of Aβ [70]. CAPN1, GSK3β and CDK5 markedly increase hyperphosphorylation of tau [71]. This study showed that these target proteins are important factors in the therapeutic effects of GF on AD. Moreover, these targets are involved in the two important pathological features of AD.

CDK5 was reported to be associated with the pathogenesis of AD and exhibited severe toxicity to neurons [72]. Additionally, the activity of CDK5 enhanced the accumulation of Aβ [73] and mediated tau hyperphosphorylation [74, 75]. However, the association between CDK5 and GF has not been sufficiently explored. In this study, we verified CDK5 expression in the control and AD groups in a GEO dataset and found that CDK5 expression was decreased in the entorhinal cortex, hippocampus and temporal cortex of AD patients. CDK5 mRNA expression was reduced by GK in the present study, indicating that CDK5 might be an anti-AD target of GF. GSK3β is a primary tau kinase that is most strongly implicated in tau pathology in AD [76]. A previous study in animals confirmed that GF inhibited the activity of GSK3β in transgenic mice expressing the human P301S tau mutant to prevent AD pathogenesis [77]. The results of our GEO dataset analysis showed that GSK3β expression was decreased in the temporal cortex of AD patients. However, the mRNA and protein levels of GSK3β were not changed. We further detected p-GSK3β-S9 (inactivated form) through Western blotting and found that its level was increased with GK treatment, indicating that GSK3β promotes AD pathogenesis as a therapeutic target of GF. Therefore, it is important to explore the pathological changes in AD. A previous study showed that BACE1 initiates amyloidogenic processing of APP, which eventually results in synthesis of Aβ [78]. BACE1 catalyzes the initial cleavage of APP to generate Aβ; therefore, inhibition of BACE1 activity could prevent the earliest pathological events in AD. However, GF did not affect BACE1 activity in cultured neurons or in Tg2576 mice [79]. Molecular docking results showed that GK has a strong binding effect with GSK3β, BACE1 and CDK5. In this study, we verified that the neuroprotective effect of GK in AD is related to mediation of GSK3β and CDK5.

To summarise, this study identified the key ingredients, core targets and pathways of GF in treating AD through a pharmacological approach. The protective effect of GK against AD pathogenesis was verified in N2A-APP cells. Our evidence indicated that the potential mechanism by which GF ameliorates multiple pathological features of AD was direct synergy among effects on multiple targets and pathways. These results provide evidence supporting the clinical application of GK in AD treatment.

## MATERIALS AND METHODS

### Drugs and antibodies

GK (purity ≥ 98%, # 437-64-9) was obtained from MedChemExpress (Shanghai, China) and dissolved in DMSO. Antibodies specific for β-actin (# 60008-1-Ig), total GSK3β (t-GSK3β) (# 22104-1-AP) and were purchased from Proteintech (Wuhan, China). An antibody specific for GSK3β phosphorylated at Ser9 (p-GSK3β) was from Cell Signaling (Danvers, MA, USA). Antibodies specific for CDK5 (# ab40773) was from Abcam (Abcam, Cambridge, UK). Anti-rabbit (# 926-32210) and anti-mouse IgG (# 926-32211) conjugated to IRDye^®^ 800 CW used for Western blotting were purchased from Li-Cor Bioscience (Lincoln, NE, USA).

### Screening of the main active ingredients and targets of GF

All of the ingredients of GF were collected from the most recent version of the TCMSP database (updated in June 2, 2021) [32] (https://tcmspw.com/tcmsp.php). The screening criteria are OB ≥ 30%, DL score ≥ 0.18 and a BBB score ≥ –0.3. The compounds with BBB score ≥ –0.3 readily cross the BBB. The SwissADME web tool (http://www.swissadme.ch) was used to evaluate the Lipinski's Rule of Five of the main active ingredients of GF. SwissTargetPrediction [33] (http://www.swisstargetprediction.ch/) was employed to identify the ingredient-related target proteins based on the determined main active ingredients.

### Collection of AD targets

AD targets were collected from the GeneCards database (https://www.genecards.org/), DrugBank database (https://go.drugbank.com/), TTD (http://db.idrblab.net/ttd/) [34] and Chemogenomics Database for Alzheimer's Disease (https://www.cbligand.org/AD/mainpage.php) using “Alzheimer's disease” as the keyword. Duplicate targets were removed using Microsoft Excel 2019 software (Redmond, WA, USA).

### Network of the main active ingredients-AD target

The overlaps between GF and AD targets were generated with Venny 2.1 (https://bioinfogp.cnb.csic.es/tools/venny/index.html), and the main active ingredient-AD target network was constructed using Cytoscape (version 3.7.1) [80]. The active ingredient nodes are colored red, and target protein nodes are colored blue.

### PPI network construction and screening of core targets

The PPI network for the activity of GF against AD was constructed using STRING database version 11.0 (https://string-db.org/) [35]. The organism was set to *Homo sapiens*, and only interactions meeting the criterion of a minimum required interaction score > 0.4 were considered significant. The PPI network comprises nodes, which represent target proteins, and edges, which represent protein interactions. The degree refers to the number of other nodes directly connected to a node. The higher the degree, the more important is the node. Core targets were identified through network analysis using Cytoscape software and its plugin (NetworkAnalyzer). In addition, target proteins were categorized with the Panther classification system (http://pantherdb.org/) [81]. Herbs contains GK were obtained from HERB database (http://herb.ac.cn/).

### GO and KEGG pathway enrichment analyses

GO biological process and KEGG pathway enrichment analyses were performed using Metascape (https://metascape.org/gp) [36]. Enriched terms with *p* < 0.01, a minimum count of 3, and an enrichment factor > 1.5 were considered significant. An online tool (http://www.bioinformatics.com.cn) was used to visualize the top 20 enriched terms. In addition, a target-KEGG pathway network for the activity of GF against AD was constructed with Cytoscape (version 3.7.1).

### Analysis of GF targets related to AD pathology

AlzData (http://www.alzdata.org/) [37] is an AD-related database that collects current high-throughput omics data. Gene symbols of the anti-AD target proteins of GF were used as input into AlzData for correlation analysis with AD pathology (Aβ and tau). Microsoft Excel 2019 software was then used to collate the obtained results. The normalized expression levels of AD-associated GF targets anti-AD targets of GF in the control and AD groups in the GEO dataset were analyzed with the “Differential expression” module of AlzData. GraphPad Prism software (version 8.0) was used for graphical visualization. ROC curves were plotted using GraphPad Prism software.

### Molecular docking

The chemical structures of GK were downloaded from the PubChem database (https://pubchem.ncbi.nlm.nih.gov/) [82]. The crystal structure was accessed from the RCSB PDB protein data bank (PDB, http://www.rcsb.org/pdb/). The molecular docking procedure was performed according to the protocol within LeDock (http://www.lephar.com/software.htm) [39]. The interaction of residues between GK and targets was analyzed by LigPlot (https://www.ebi.ac.uk/thornton-srv/software/LIGPLOT/) [40].

### Cell culture

N2A-APP cell line was kindly gifted by Dr. Pei Jin-jing (Karolinska Institute, Stockholm, Sweden) [41]. Cells were cultured in DMEM supplemented with 10% FBS and penicillin/streptomycin (DMEM/10% FBS). At the end of the experiment, the cells were rinsed twice in ice-cold PBS and lysed with buffer containing 2 mM EGTA, 0.5 mM PMSF, 5 mM EDTA, 150 mM NaCl, 50 mM Tris–HCl (pH 7.4), 1% Triton X-100, and protease inhibitor cocktail (1:100) prior to sonication for 15 s on ice.

### CCK-8 assay

Cell viability was assessed indirectly by a CCK-8 assay. N2A-APP cells were plated in 96-well plates at a suitable density. After exposing the cells to GK at different concentrations (0, 12.5, 25, 50, 100, 200, 400 μM) for 48 h, the medium was replaced with 100 μL of fresh medium containing 10% CCK-8 reagent (Dojindo Laboratories, Kumamoto, Japan) for 30 min at 37°C. Then, the absorbance values of the wells at 450 nm were measured in a BioTek Synergy 2 microplate reader (Winooski, VT, USA). Please note that the N2A-APP cells treated with 0.1% DMSO solvent served as controls in this study.

### RNA extraction and RT-PCR

RNA was isolated from renal tissues with TRIzol reagent and resuspended in sterile water. The total RNA concentration was assessed using the absorbance readings at 260 and 280 nm. RT-PCR was performed using Superscript II Reverse Transcriptase (Invitrogen) according to the instructions from the manufacturer, and PCR was conducted using the primers described previously [83]. In this study, the fold change was calculated as 2 to the ^−ΔΔCt^ power (2^−ΔΔCt^).

The following primers were used: β-actin, forward: GAGACCTTCAACACCCCAGC, reverse: GGAGAGCATAGCCCTCGTAGAT. CDK5, forward: CCCTACCCAATGTACCCAGC, reverse: GAGAAGTAGGGGTGCTGCAA. GSK3β, forward: CAGCAGCCTTCAGCTTTTGG, reverse: AACTGACTTCCTGTGGCCTG.

### Western blotting

Western blotting was performed as previously described [84–85]. The quantification of the Western blot was conducted using ImageJ (NIH, Bethesda, MD, USA)

### Statistical analysis

The data are presented as means ± SEM. Statistical analyses were performed using SPSS 19.0 statistical software (SPSS, Chicago, IL, USA), and visualized with GraphPad Prism software (GraphPad Software, Inc., La Jolla, CA). Either one-way ANOVA followed by Tukey’s multiple comparisons test or an independent-samples *t*-test was used to determine differences between groups. A significance value of *p* < 0.05 was set.

## Supplementary Materials

Supplementary Table 1
