# Supplementary material for: A network pharmacology approach to uncover the key ingredients in Ginkgo Folium and their anti-Alzheimer’s disease mechanisms
Source: Aging (Albany NY). 2021 Jul 27;13(14):18993–9012. doi: 10.18632/aging.203348 (PMC8351672; doi:10.18632/aging.203348)
Supplement: Supplementary Table 1 [file aging-13-203348-s001.pdf]

SUPPLEMENTARY TABLE

Supplementary Table 1. Main herbs containing genkwanin.

Herb id	Herb Pinyin name	Herb English name
HERB006632	YIN XING YE	*Ginkgo Folium*
HERB000253	BAI MU XIANG	*Chinese Eaglewood*
HERB000508	BO HE	*Mentha, Peppermint*
HERB000910	CI CAO SU	*Pungent Jerusalemsage*
HERB001032	DA FENG ZI	*Hydnocarpi Anthelminticae Semen*
HERB001407	DU YI WEI	*Common Lamiophlomis*
HERB002000	GUANG GUO GAN CAO	*Licorice*
HERB002002	GUANG HUO XIANG	*Pogostemon Cablin (Blanco) Benth.*
HERB002658	JI CHA KAI LA RUI A	*Spreading Creosote-bush*
HERB002870	JIAO ZHI SHU WEI CAO	*Sticky Clary*
HERB002991	JING QIAO MAI	*Rhizome fagopyri dibotryis*
HERB003282	LE GE WANG GEN	*Wikstroemiae Indicae Rasix*
HERB003658	MA HUANG	*Ephedra*
HERB003917	MI DIE XIANG	*Rosemary*
HERB004546	QING YE DAN	*All - grass of Mile Swertia*
HERB004757	SAN CHI LA RUI A	*Creosote-bush*
HERB004950	SHAN ZHU YU	*Asiatic Cornelian Cherry Fruit*
HERB004973	SHE GAN	*Blackberrylily Rhizome*
HERB005039	SHI DI	*Calyx and receptacle of a Persimmon*
HERB005132	SHU QU CAO	*Gphalii Affinis Herba*
HERB005412	TAO REN	*Peach Seed*
HERB005915	XI YE YI MU CAO	*Siberian Motherwort*
HERB006537	YI MU CAO	*Motherwort*
HERB006586	YIN CHEN	*Virgate Wormwood Herb*
HERB006587	YIN CHEN HAO	*Capillary Wormwood Equivalent plant: Artemisia scoparia*
HERB006655	YING TAO	*Falsesour Cherry*
HERB006718	YU JIN	*Tuber - root of Common Turmeric*
HERB006773	YUAN HUA	*Flower bud of Lilac Daphne*
HERB006881	ZAO XIN TU	*Earth inside of Hearth*
